# The Autoimmune-Associated Single Nucleotide Polymorphism Within *PTPN22* Correlates With Clinical Outcome After Lung Transplantation

**DOI:** 10.3389/fimmu.2018.03105

**Published:** 2019-01-17

**Authors:** Kevin Budding, Jessica van Setten, Eduard A. van de Graaf, Oliver A. van Rossum, Tineke Kardol-Hoefnagel, Johanna M. Kwakkel-van Erp, Erik-Jan D. Oudijk, C. Erik Hack, Henderikus G. Otten

**Affiliations:** ^1^Laboratory of Translational Immunology, University Medical Center Utrecht, Utrecht, Netherlands; ^2^Department of Cardiology, University Medical Center Utrecht, Utrecht, Netherlands; ^3^Department of Respiratory Medicine, University Medical Center Utrecht, Utrecht, Netherlands; ^4^Center of Interstitial Lung Diseases, St. Antonius Hospital, Nieuwegein, Netherlands; ^5^Departments of Rheumatology and Dermatology, University Medical Center Utrecht, Utrecht, Netherlands

**Keywords:** lung transplantation, PTPN22, chronic lung allograft dysfunction, bronchiolitis obliterans syndrome, transplantation genetics

## Abstract

Obstructive chronic lung allograft dysfunction (BOS) is the major limiting factor for lung transplantation (LTx) outcome. *PTPN22* is described as the hallmark autoimmunity gene, and one specific single nucleotide polymorphism (SNP), rs2476601, is associated with multiple autoimmune diseases, impaired T cell regulation, and autoantibody formation. Taking into consideration the contribution of autoimmunity to LTx outcome, we hypothesized that polymorphisms in the *PTPN22* gene could be associated with BOS incidence. We selected six SNPs within *PTPN22* and analyzed both patient and donor genotypes on BOS development post-LTx. A total of 144 patients and matched donors were included, and individual SNPs and haplotype configurations were analyzed. We found a significant association between patients carrying the heterozygous configuration of rs2476601 and a higher risk for BOS development (*p* = 0.005, OR: 4.400, 95%CI: 1.563–12.390). Kaplan-Meier analysis showed that heterozygous patients exhibit a lower BOS-free survival compared to patients homozygous for rs2476601 (*p* = 0.0047). One haplotype, which solely contained the heterozygous risk variant, was associated with BOS development (*p* = 0.015, OR: 7.029, 95%CI: 1.352–36.543). Our results show that LTx patients heterozygous for rs2476601 are more susceptible for BOS development and indicate a deleterious effect of the autoimmune-related risk factor of *PTPN22* in patients on LTx outcome.

## Introduction

Lung transplantation (LTx) is the last line of treatment for patients suffering from end-stage lung diseases including cystic fibrosis (CF), chronic obstructive pulmonary disease (COPD), and interstitial lung disease (ILD). LTx is the least successful solid organ transplantation due to the high incidence of chronic lung allograft dysfunction (CLAD). CLAD is clinically defined as obstructive (bronchiolitis obliterans syndrome, BOS), the most observed form of CLAD, or restrictive (restrictive allograft syndrome, RAS) (1). Latest numbers show that 5 year BOS-free survival is 50%, whereas only 25% of the transplanted patients remain free from BOS within the first 10 years after LTx (2).

BOS is a heterogeneous complication in which multiple parts of the immune system appear to play a role. Evidence exist for the involvement of humoral immunity, including complement and antibody formation, and features of autoimmunity (3). Also, cellular mediated processes, in which T cell reactivity seems to play a pivotal role, have been observed in patients diagnosed with BOS (4). A remarkable example of antibody formation is the association between autoantibodies directed against K-alpha 1 tubulin and collagen V and the development of BOS, also in the absence of donor specific anti-HLA antibodies (5). Furthermore, auto reactive CD4+ T cells directed against collagen V are observed in LTx patients, indicating that both B cell and T cell mediated autoimmunity plays a pivotal role in BOS development (6).

Upon T cell receptor-ligand interaction, T cell reactivity is controlled, among others, by intracellular proteases and phosphatases. One important protein tyrosine phosphatase (PTP) involved in T cell regulation is encoded by the *PTPN22* gene, which is located on chromosome 1. The *PTPN22* gene is considered to be one of the most important autoimmunity risk genes. Within the *PTPN22* gene different single nucleotide polymorphisms (SNPs) are present, of which rs2476601 is the most striking clinically relevant example. Autoimmunity-related associations are, among others, rheumatoid arthritis (RA) (7), systemic lupus erythematosus (SLE) (8), vitiligo (9), and progressive systemic sclerosis (10, 11). The SNP rs2476601 is a missense mutation leading to an arginine tryptophan substitution within the first proline-rich sequence of the C-terminal domain, resulting in a disrupted interaction with CSK and enhanced enzyme activity (12). Currently, different functional models for the PTPN22–R620W substitution are described in literature (13).

Besides investigations on associations in autoimmunity and functional implications on T cell receptor signaling, *PTPN22* SNPs are also studied in the field of transplantation. In kidney transplantation, *PTPN22* gene polymorphisms were studied in two independent cohorts, but no associations with kidney function could be observed after transplantation (14, 15). Interestingly, Dullin et al. investigated selected SNPs within the *PTPN22* gene and observed that rs2476601 is associated with multiple episodes of acute rejection after liver transplantation (16).

Taking into consideration the results obtained in liver transplantation, the associations of PTPN22 with autoimmunity and the observed features of autoimmunity in LTx, we hypothesized that SNPs in the *PTPN22* gene could be associated to outcomes after LTx. For this end, we selected 6 SNPs within the *PTPN22* gene and used both genotype and haplotype analysis on patient and donor samples and assessed correlations with transplantation outcome.

## Patients and Methods

### Patients

For this study, we included 144 patients and their respective donors that were treated with LTx between January 2004 and 2013. From al study participants written informed consent was obtained and this study was approved by the medical ethical committee of the University Medical Center Utrecht (METC 06-144). All patients received standardized immunosuppressive therapy consisting of tacrolimus, prednisolone, and mofetil mycophenolate. Furthermore, patients received treatment with valganciclovir for up to 6 months when categorized as at high risk for cytomegalovirus (CMV) or Epstein-Barr virus (EBV), defined as a CMV- or EBV-negative patient receiving a graft from a CMV- or EBV-positive donor. BOS was diagnosed according to international guidelines as a decline of the forced expiratory volume in 1 s in absence of any other cause of disease of 20% compared to baseline level (17). Samples of donor spleen, donor blood, and patient blood were collected prior to or during transplantation procedure. From each respective sample, peripheral blood mononuclear cells were isolated using Ficoll-paque gradient centrifugation (20 min, 2400 rpm, break/acceleration 2) and stored in liquid nitrogen until further analysis.

### DNA Isolation, Genotyping, and SNP Selection

The MagnaPure Compact System (Roche Diagnostics, Basel, Switzerland) was used for DNA isolation from frozen PBMC samples, according to manufacturer's instructions.In addition, cell samples were thawed at 37°C and dissolved in RPMI-1640 (Lonza, Basel, Switzerland) 20% fetal bovine serum (Bodinco, Alkmaar, The Netherlands), followed by 10 min centrifuging at 1,800 rpm. The obtained cell pellet was dissolved at a concentration of 5 × 10^6^ cells/ml in phosphate buffered saline, and used for DNA isolation. DNA samples were included when DNA purity ratios were between 1.7 and 2.1 for 260/280 and between 1.2 and 2.1 for 260/230. Samples were genotyped within the *i*GeneTRA*i*N network using the specifically designed and developed Affymetrix “TxArray,” which contains 767,203 variants. Briefly, the array is designed around ~350 k genome-wide coverage markers supplemented with module-specific content from the UK Biobank core array, transplant-specific content, and a GWAS booster module for optimal coverage for whole genome imputation. An extensive overview of the TxArray and validation studies can be found in dedicated articles (18, 19). Subsequently, samples were subjected to stringent quality control (QC) in order to remove both low-quality genotyped SNPs and samples. We removed samples with a missing rate >3%. Subsequently, we generated a subset of high-quality independent SNPs according to the following conditions: missing rate <1%, Hardy-Weinberg *P* > 0.001, minor allele frequency >0.1 and LD pruning leaving no SNP-pairs with *r*^2^ > 0.2. Subsequently, samples were removed with heterozygosity >2SD from the mean of all samples, related samples (keeping only one samples of each pair with proportion of IBD >0.2), and samples of non-European ancestry based on principle component analysis using the 1000 Genomes Project (Phase 1) populations as reference (20). SNPs were removed if they presented a missing rate >5%, Hardy-Weinberg *P* < 0.01, or when they were monomorphic. After QC, 543,637 SNPs and 132 patients and 131 donor samples remained. Untyped SNPs were imputed (21, 22) with the 1,000 Genomes Project (v3) (23) and the Genomes of the Netherlands (v5) (24) as reference panels. Samples were phased with SHAPEIT (25) and imputed with IMPUTE v2 (26). Sequence data has been deposited at the European Genome-phenome Archive (EGA), which is hosted by the EBI and the CRG, under accession number EGAS00001003380. Further information about EGA can be found on https://ega-archive.org “The European Genome-phenome Archive of human data consented for biomedical research” (http://www.nature.com/ng/journal/v47/n7/full/ng.3312.html).

Six SNPs in the *PTPN22* gene that were frequent in the Western European population were selected. Furthermore, these SNPs were selected according to published literature on PTPN22-related transplantation research (14–16), and their associations with other disease phenotypes. SNP rs2488457 lies in the promotor region of *PTPN22* within the binding site for the transcription factor activator protein 4, and is associated with protein expression levels (27). SNP rs33996649, located in exon 10, is protective in SLE (28), whereas rs2476601 is one of the most important associated genetic polymorphism associated with autoimmunity (29). Both rs1310182 and rs1217388 are located within intronic transcription factor binding sites, and rs3789604 is a downstream variant, which is located in a transcription factor binding site implicated in protein expression (30).

### Statistics

We used SPSS version 21 (IBM Corp., Armonk, NY), GraphPad Prism version 6.02 (GraphPad Software Inc., San Diego, CA) and R version 3.0.3 (The R Foundation for Statistical Computing, Vienna, Austria) for statistical analyses. Categorical data were analyzed via the Fischer's exact test, whereas differences in continuous variables were assessed via ANOVA. Odds ratios (OR) and 95% confidence intervals (CI) were generated via logistic regression and used to estimate strengths of associations. Kaplan-Meier analyses were used for survival analyses and differences were analyzed via log-rank test. Cox regression was used for multiple predictors. After Bonferroni correction for 6 variables a *p*-value < 0.008 was considered to be statistically significant.

## Results

### Patient and Donor Demographics

A total of 144 LTx patient and donor couples were included in this study, based upon material availability. Sixty-five of these patients were transplanted because of chronic obstructive pulmonary disease, 42 because of cystic fibrosis, 36 due to interstitial lung disease and one patient was diagnosed with pulmonary vascular disease as primary lung disease. Forty-four patients developed BOS during follow-up and RAS was not observed. Patient and donor clinical and demographical parameters are depicted in Table 1. No significant differences were observed between BOS+ and BOS- patients, except for the mean age at the time of transplantation, which was slightly higher in the BOS+ patient group. During follow-up, 44 patients deceased and a total of 20 patients presented with either one or more episodes of acute rejection.

**Table 1:** Patient and donor demography.

	**All**	**No BOS**	**BOS**	***p***
**PATIENTS**				
Total number	144	100	44	
**GENDER**				
Male	69	48	21	0.560
Female	75	52	23	
Mean age (years)	46 ± 13	44 ± 14	50 ± 11	*0.026*
Mean follow-up (months)	61.2 ± 36.8	59.2 ± 39.4	65.6 ± 30.2	0.341
**PRIMARY DISEASE**				
COPD	65	40	25	0.247
CF	42	33	9	
ILD	36	26	10	
PVD	1	1	0	
**Infection**				
EBV high risk	14	7	7	0.115
CMV high risk	32	21	11	0.456
**Type of graft**				
Bilateral	112	81	31	0.119
Single	32	19	13	
Episode of acute rejection	20	14	6	0.495
**Ischemic times (min)**				
Bilateral	312.3 ± 188.9	321.4 ± 216.9	288.6 ± 73.8	0.426
Single	244.1 ± 53.5	238.2 ± 48.8	238.7 ± 73.0	0.314
**DONORS**				
**Gender**				
Male	65			
Female	79			
**Donor age (years)**				
Mean age	45 ± 14			
>60	17			
**Smoking**				
Yes	52			
No	92			
**Donor type**				
HB	116			
non HB	28			

*BOS, bronchioltis obliterans syndrome; COPD, chronic obstructive pulmonary disease; CF, cystic fibrosis; ILD, Interstitial lung disease; PVD, pulmonary vascular disease; EBV, Epstein-Barr virus; CMV, cytomegalovirus; HB, heart beating; NHB, non-heart beating. Significant values are depicted in italics*.

### *PTPN22* Single Nucleotide Polymorphism Distribution

The selected *PTPN22* SNPs, see Patients and Methods, were genotyped via imputation and both SNPs and samples were subjected to stringent quality control steps (31). A total of 132 patients, consisting of 41 patients who did develop BOS and 91 patients who did not, passed the sample quality control steps. For the donors these numbers were slightly different; 131 samples passed quality control check and 39 coupled patients developed BOS during follow-up and 92 did not. Total genotyping results and SNP frequencies are depicted in Table 2 for both patients and donors, stratified per diagnosis of chronic rejection. Selected SNPs were tested for association with chronic rejection in both patients and donors. We observed an association between the SNP configuration of rs2476601 in patients, but not in donors, and the incidence of BOS during follow-up after LTx. The frequency distribution of the heterozygous variant (G/A) was increased in BOS+ compared to BOS- patients (27 vs. 8%, respectively). This association was significant and patients carrying this heterozygous genotype present with an increased risk for BOS development (*p* = 0.005, OR: 4.400, 95% CI: 1.563–12.390). No association between either genotype in patients or donors was observed with episodes of acute rejection (data not shown).

**Table 2:** Genotype frequencies per *PTPN22* SNP for both lung transplantation recipients and donors.

		**Patient**					**Donor**		
		**No BOS**	**BOS**				**No BOS**	**BOS**	
**SNP**		***n* = 91**	***n* = 41**	***p***	**OR**		***n* = 92**	***n* = 39**	***p***
rs2488457	GG	5 (5%)	5 (12%)	NS		GG	6 (7%)	2 (5%)	NS
	GC	26 (29%)	12 (29%)			GC	32 (35%)	11 (28%)	
	CC	60 (66%)	24 (59%)			CC	54 (59%)	26 (67%)	
rs33996649	CC	88 (97%)	40 (98%)	NS		CC	85 (92%)	36 (92%)	NS
	CT	3 (3%)	1 (2%)			CT	7 (8%)	3 (8%)	
	TT	0 (0%)	0 (0%)			TT	0 (0%)	0 (0%)	
rs2476601	GG	82 (90%)	30 (73%)	0.005	4.400 (1.563–12.390)	GG	74 (80%)	31 (79%)	NS
	GA	7 (8%)	11 (27%)			GA	15 (16%)	7 (18%)	
	AA	2 (2%)	0 (0%)			AA	3 (3%)	1 (3%)	
rs1310182	AA	20 (21%)	9 (22%)	NS		AA	19 (21%)	3 (8%)	NS
	AG	44 (48%)	19 (46%)			AG	43 (47%)	21 (54%)	
	GG	27 (30%)	13 (32%)			GG	30 (33%)	15 (38%)	
rs1217388	GG	6 (7%)	0 (0%)	NS		GG	8 (9%)	2 (5%)	NS
	GA	27 (30%)	32 (78%)			GA	34 (37%)	13 (33%)	
	AA	58 (64%)	9 (22%)			AA	50 (54%)	24 (62%)	
rs3789604	TT	52 (57%)	28 (68%)	NS		TT	62 (67%)	30 (77%)	NS
	TG	32 (35%)	13 (32%)			TG	28 (30%)	8 (21%)	
	GG	7 (8%)	0 (0%)			GG	2 (2%)	1 (3%)	

*BOS, bronchiolitis obliterans syndrome; OR, odds ratio +95% confidence interval, obtained via logistic regression analysis; NS, not significant*.

*Percentages approximated. Donors were stratified according to BOS incidence in the respective recipient*.

### Heterozygosity for rs2476601 Is Associated With Lower BOS-Free Survival Post-LTx

**Figure 1:**
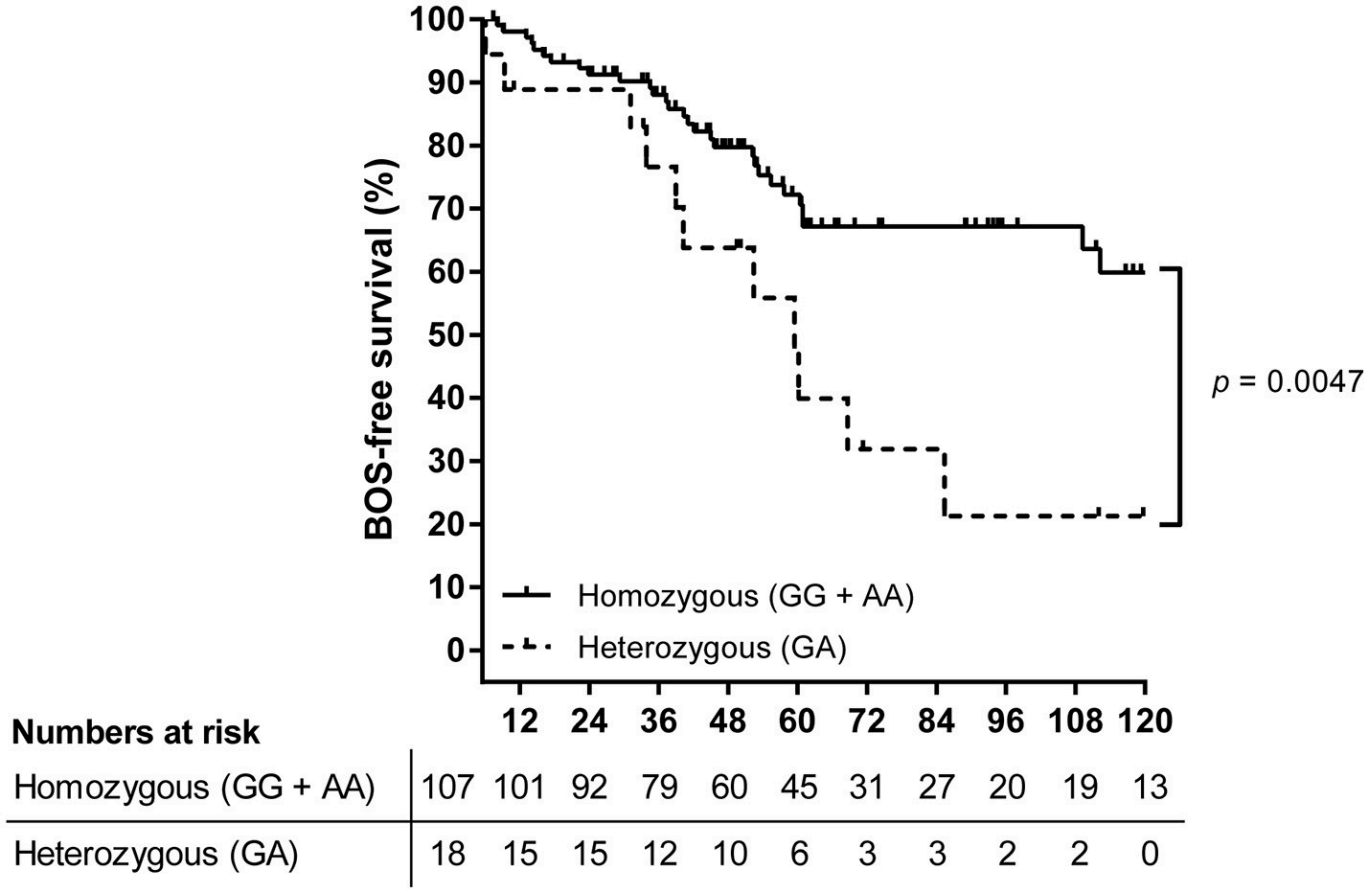
Survival analysis on BOS incidence in LTx patients stratified per rs2476601 genotype. A total of 125 patients were stratified according to rs2476601 genotype, either as heterozygous (GA) or homozygous (GG and AA). Patients who deceased within the first 6 months after transplantation were excluded from analysis. The numbers represent patients at risk for BOS development during follw-up. Patients genotyped as heterozygous (dashed line) present a lower BOS-free survival rate compared to homozygous patients (solid line), *p* = 0.0047, log-rank test.

The primary observation that LTx patients genotyped heterozygous for SNP rs2476601 are at higher risk for BOS development post-LTx was confirmed via Kaplan-Meier and subsequent log-rank analysis. After the exclusion of patients who deceased within the first 6 months post-LTx, 125 patients were included. Kaplan-Meier analysis showed that patients heterozygous for rs2476601 have a significant lower BOS-free survival rate compared to homozygous patients (Figure 1, *p* = 0.0047).

### Haplotype Analyses on *PTPN22* SNPs

Stratification of the identified configurations of the selected *PTPN22* SNPs resulted in the generation of 19 different haplotypes (frequencies from 29% till 0.8%) in the patient group and 17 different haplotypes (frequencies from 30% till 0.8%) in the donor group (Table 3). We analyzed five patient and four donor haplotypes with a frequency >5% in our cohort. The high-risk heterozygous configuration of rs2476601 was present in only one haplotype, which was associated with BOS (*p* = 0.015, OR: 7.029, 95% CI: 1.352–36.543).

**Table 3:** Patient and donor haplotype analysis on BOS incidence after LTx.

	**Haplotype**	**1**	**2**	**3**	**4**	**5**
	rs2488457	C	C	S	S	C
	rs33996649	C	C	C	C	C
	rs2476601	G	G	G	R	G
	rs1310182	G	R	R	R	A
	rs1217388	A	A	R	R	A
	rs3789604	T	K	T	T	G
Patient	Frequency	38 (29%)	31 (23%)	17 (13%)	8 (6%)	7 (5%)
	No BOS	26 (29%)	22 (24%)	14 (15%)	2 (2%)	7 (8%)
	BOS	12 (29%)	9 (22%)	3 (7%)	6 (15%)	0 (0%)
	*p*	NS	NS	NS	0.015	NS
Donor	Frequency	39 (30%)	24 (18%)	15 (12%)	15 (12%)	
	No BOS	26 (28%)	16 (17%)	11 (12%)	9 (10%)	
	BOS	13 (33%)	8 (21%)	4 (10%)	6 (15%)	
	*p*	NS	NS	NS	NS	

*BOS, bronchiolitis obliterans syndrome; NS, not significant*.

*Percentages approximated*.

## Discussion

The *PTPN22* gene is considered to be the most important non-HLA autoimmunity gene, of which the genetic variant rs2476601, 1858 C>T leading to an arginine–tryptophan substitution, is associated with multiple indications of autoimmunity including RA, SLE, and systemic sclerosis. Given the reported associations between autoimmunity and outcome after LTx we assessed the relation of the *PTPN22* risk variant in both patients and donors and the incidence of chronic rejection after LTx.

Features of autoimmunity are often observed in or associated with chronic rejection after LTx (32). Type V collagen and K-α 1 tubulin are antigens for autoantibodies, presumably due to tissue remodeling and the activity of matrix metalloproteases that are induced by ischemia reperfusion injury and cleave collagen, thereby releasing antigenic fragments. Binding of autoantibodies to their respective targets on airway epithelial cells results in increases of profibrotic growth factors and proinflammatory cytokines (5, 33). Linked-recognition requires antigen recognition by T cells, and indeed auto reactive T cells directed at Type V collagen are present in LTx patients (6).

The non-receptor PTP encoded by *PTPN22* consists of different domains, including the N-terminal catalytic active domain involved in dephosphorylation and a C-terminal domain which consists of proline-rich regions that facilitate binding of intracellular adapter proteins (29). PTPN22 is exclusively expressed by hematopoietic cells (34), and distinctive roles have been described in the regulation of immune cell signaling (13, 35). In T cell signaling, PTPN22 inhibits T cell activation via inhibiting downstream T-cell receptor signaling. In more detail, T cell activation after T cell receptor binding requires a cascade of tyrosine phosphorylation steps involving Src- (LCK, Fyn), and Syk family (ZAP-70) member kinases, that phosphorylate immunoreceptor tyrosine-based activation motifs located on the intracellular CD3-ζ chain (36). These all function as substrates for PTPN22, resulting in the dephosphorylation of their respective activation, and inhibition of T cell signaling (37). Furthermore, in T cells, the adapter protein binding domains on PTPN22 mediate the binding of tyrosine-protein kinase CSK, which also inhibits intracellular T cell receptor signaling (38, 39).

*PTPN22* rs2476601 is associated with the occurrence of autoantibodies in RA, including rheumatoid factor and anti-citrullinated peptide (7), presumably due to the contributive effect on the generation on autoreactive B cells. Dai et al. have demonstrated spontaneous autoimmunity with infiltrations in both lungs and livers in a C57BL/6x129 mouse model homologous to the human PTPN22 risk variant (40). Also, deregulation in the deletion of clonal B cells and escape of autoreactive B cells from deletion stimuli have been observed in studies concerning the PTPN22 risk variant (41). These observations suggest a contributing factor of rs2476601 in autoantibody production, associated with impaired clinical outcome after LTx.

T regulatory cells are known to induce tolerance to self-antigens and suppress Th-1 autoimmunity, which has also been shown in human LTx (6). An altered T cell differentiation model is introduced as explanation for the pathogenesis toward autoimmunity. Disbalance between T cells and T regulatory cells is observed in *PTPN22* knockout mice, suggesting tight regulation between effector and regulatory T cells via PTPN22. This has been strengthened by research of Vang et al. showing that T cells from individuals homozygous for the *PTPN22* risk variant depicted increased Th-1 induced IFN-γ secretion and a decreased suppression of Th-1 cells via regulatory T cells (42). Translating these observations to the field of LTx suggests that a decreased suppression of Th-1 immune responses in patients genotyped with the risk variant of PTPN22 could contribute to autoimmunity and the pathogenesis of BOS development.

Here, we analyzed six selected *PTPN22* gene polymorphisms in a cohort of patients who underwent LTx. Also, coupled donors were analyzed for these specific SNP configurations. Our analysis shows that a single missense SNP in the *PTPN22* gene in patients is associated with outcome after LTx and the incidence of obstructive chronic lung allograft dysfunction. From our Kaplan-Meier analysis we can conclude that this association is predominantly with late chronic allograft dysfunction, indicated by the difference in inclination after month 30 post-LTx. Of the analyzed haplotypes only one haplotype showed a significant association with BOS incidence. However, this haplotype was only present in a relatively small number of BOS patients (15%), and validation of this finding is imperative.

In this study we have not been able to include an auto-antibody screening for anti-collagen V or anti-K-alpha 1 tubulin antibodies. However, our group has previously identified autoantibodies against ETAR, AT1R and BPIFA1/SPLUNC1 in pre- and post-LTx sera of the investigated cohort. Anti-ETAR and anti-AT1R autoantibodies are correlated with kidney transplantation outcome and anti-BP1FA1/SPLUNC1 autoantibodies are present in patients with endstage CF (43, 44). We found no correlation between autoantibody titers or stratified rs2476601 SNP configuration (data not shown). Within our cohort, we observed differences between the age at the time of LTx procedure between BOS+ and BOS− patients. Patients developing BOS were significantly older compared to non-BOS patients. This differs from other studies, were the reverse was observed, explained by the fact that younger patients have a less active immune system, although this remains purely speculative (45, 46). After Cox regression modeling in our cohort, including both the risk allele for *PTPN22* and age at time of LTx, SNP rs2476601 was still an independent risk factor for BOS development (*p* = 0.004, OR: 2.748, 95% CI: 1.369–5.516, data not shown).

Targeted SNP analyses and genetic studies focused on solid organ transplantation and especially LTx outcome have increased over the past few years (47). Our group previously reported a genetic promotor polymorphism in complement regulation to be associated with chronic rejection after LTx, with the rationale that after binding to the graft, antibodies are less functional (48). Furthermore, the LTx research group from Leuven recently published findings on a polymorphism in the IL-17 receptor that predisposes to primary graft dysfunction (49), and correlations between SNP genotypes and immunosuppressive therapy efficacy have been described (50). These results are encouraging and indicate the importance of this emerging field, despite the need of prospective multicenter and validation studies. The recently installed aforementioned *i*GeneTRA*i*N consortium could facilitate these needs (18, 19).

Studies concerning the influence of rs2476601 on transplantation outcome have been conducted on other solid organ transplantations, albeit with different results. In liver transplantation, associations between rs2476601 and multiple episodes of acute rejection were observed, although chronic rejection associations were not assessed (16). No associations with outcome after kidney transplantation and *PTPN22* gene polymorphisms were found by two independent groups (14, 15). Inter-organ differences could be explained by variations in either humoral or cellular immunologic mechanisms predisposing chronic rejection pathology, or differences in immunosuppressive regimens (51).

To summarize, our results are the first to show a deleterious effect of the autoimmune-related risk factor of *PTPN22* in patients on LTx outcome. These results could be used for the further optimization of risk stratification algorithms to identify LTx outcome prior to, or earlier after transplantation. More research is expedient to elucidate the specific pathogenic pathways affected by PTPN22 in the development of chronic rejection after LTx. Importantly, larger study cohorts are needed to replicate our findings and to incorporate other genetic polymorphisms, in both patients and donors, associated with the development of rejection after transplantation. Our results could serve to further optimize patient treatment and follow-up therapy, and increase the long-term efficacy of LTx treatment for selected end-stage lung disease patients.

## Author Contributions

KB, JvS, TK-H, and OvR performed the research. KB, JvS, EvdG, OvR, CH, and HO participated in data analysis. EvdG, JK, and E-JO contributed patient material. EvdG and HO participated in research design. KB, JvS, EvdG, CH, and HO wrote the paper. All authors provided final approval of the version to be published.

### Conflict of Interest Statement

The authors declare that the research was conducted in the absence of any commercial or financial relationships that could be construed as a potential conflict of interest.

## Abbreviations

BOS: Bronchiolitis obliterans syndrome
CLAD: Chronic lung allograft dysfunction
LTx: Lung transplantation
PBMC: Peripheral blood mononuclear cell
RAS: Restrictive allograft syndrome
RA: Rheumatoid arthritis
SLE: Systemic lupus erythematosus
SNP: Single nucleotide polymorphism.
